# Susceptibility to Undue Influence: The Role of the Medical Expert in Estate Litigation

**DOI:** 10.1177/07067437211020616

**Published:** 2021-06-01

**Authors:** Nathan Herrmann, Kimberly A. Whaley, Deidre J. Herbert, Kenneth I. Shulman

**Affiliations:** 1 Department of Psychiatry, 71545 Sunnybrook Health Sciences Centre, Toronto, Ontario, Canada; 2 Department of Psychiatry, University of Toronto, Toronto, Ontario, Canada; 3 Whaley Estate Litigation Partners, Toronto, Ontario, Canada; 4 McLellan Herbert, Barristers & Solicitors, Vancouver, British Columbia, Canada

**Keywords:** medico-legal issues, undue influence, testamentary capacity, geriatric psychiatry, forensic psychiatry

## Abstract

**Objectives:**

Medical experts are increasingly asked to assist the courts with Will challenges based on the determination of testamentary capacity and potential undue influence. Unlike testamentary capacity, the determination of undue influence has been relatively neglected in the medical literature. We aim to improve the understanding of the medical expert role in providing the courts with an opinion on susceptibility to undue influence in estate litigation.

**Method:**

Medical experts with experience in the assessment of testamentary capacity and susceptibility to undue influence collaborated with experienced estate litigators. The medical literature on undue influence was reviewed and integrated. The lawyers provided a historical background and a legal perspective on undue influence in estate litigation and the medical experts provided a clinical perspective on the determination of susceptibility to undue influence. Together, they provided recommendations for how the medical expert could best assist the court.

**Results:**

Susceptibility to undue influence is frequently used in estate litigation to challenge the validity of Wills and is defined as subversion of the testator’s free will by an influencer, resulting in changes to the distribution of the estate. While a determination of undue influence includes the documentation of indicia or suspicious circumstances under which the Will was drafted and executed, medical experts should focus primarily on the susceptibility of the testator to undue influence. This susceptibility should be based on a consideration of cognitive function, psychiatric symptoms, physical and behavioural function, with evidence derived from the medical documentation, the medical examination, and the history.

**Conclusions:**

The determination of undue influence is a legal one, but medical experts can help the court achieve the most informed legal decision by providing relevant information on clinical issues that may impact the testator’s susceptibility to undue influence.

## Abstract (fr)

**Objectifs:**

On demande de plus en plus souvent aux experts médicaux leur aide dans les tribunaux pour des contestations de testament basées sur la détermination de la capacité de tester et d’une influence indue potentielle. Contrairement à la capacité de tester, la détermination d’une influence indue a été relativement négligée dans la littérature médicale. Nous cherchons à améliorer la compréhension du rôle de l’expert médical qui offre aux tribunaux une opinion sur la susceptibilité à une influence indue dans un litige successoral.

**Méthode:**

Les expert médicaux expérimentés dans l’évaluation de la capacité de tester et de la susceptibilité à une influence indue ont collaboré avec des avocats spécialisés en succession. La littérature médicale sur l’influence indue a été révisée et intégrée. Les avocats ont fourni des antécédents historiques et une perspective légale sur l’influence indue dans le litige successoral et les experts médicaux ont offert une perspective clinique sur la détermination de la susceptibilité à une influence indue. Ensemble, ils ont apporté des recommandations à l’égard de la façon dont l’expert médical peut aider au mieux en cour.

**Résultats:**

La susceptibilité à une influence indue est souvent utilisée dans un litige successoral pour contester la validité des testaments et elle se définit comme étant une subversion de la volonté propre du testateur par un influenceur, ce qui produit des changements au partage de la succession. Bien que la détermination d’une influence indue comporte la documentation des indices ou des circonstances suspectes dans lesquelles le testament a été rédigé et exécuté, les experts médicaux devraient se concentrer principalement sur la susceptibilité du testateur à une influence indue. Cette susceptibilité devrait se fonder sur un examen de la fonction cognitive, des symptômes psychiatriques, de la fonction physique et comportementale, et sur des données probantes extraites de la documentation médicale, de l’examen médical et des antécédents.

**Conclusions:**

La détermination de l’influence indue est de nature légale, mais les experts médicaux peuvent aider les tribunaux à parvenir à la décision légale la mieux éclairée en offrant de l’information pertinente sur les enjeux cliniques qui peuvent influer sur la susceptibilité du testateur à une influence indue.

## Introduction

As the baby-boomer generation ages, we will witness the largest transfer of wealth in human history.^1^ This will occur in the context of increasingly complex social circumstances and family organization, as well as adverse economic circumstances for a younger generation. Most recently, this has been further complicated by the COVID-19 pandemic which has not only led to a disproportionately high death rate amongst older adults but has also forced younger generations to become more reliant on parents and grandparents for financial support because of job losses and unemployment. Accordingly, we expect an increasingly large number of challenges to Wills based on the question of testamentary capacity and/or the presence of undue influence. While the determination of testamentary capacity and undue influence are ultimately legal determinations, medical experts can help the courts understand how complicated cognitive, psychiatric and medical factors might affect capacity and make testators more susceptible to undue influence. There is a reasonable amount of literature available on the assessment of testamentary capacity including recent comprehensive guides on the role of the medical expert.^2^ Much less has been written on undue influence and this can be seen as a gap in the literature, as lawyers frequently ask medical experts to comment on this, and in some jurisdictions, Will challenges based on undue influence are even more common than challenges to testamentary capacity.^3^

Unlike testamentary capacity, which has been invariably based on criteria derived from *Banks v Goodfellow* ^4^ undue influence has not been universally defined by the courts. Historically, it has been described as external coercion or compulsion by an influencer that removes or reduces the testator’s free will and results in a change to the distribution of the estate. More recently, the notion of the subversion of will or “will substitution”^5^ has been considered the essential feature of undue influence without necessarily invoking threats or coercion. This type of definition implies that multiple factors need to be considered by the courts in order to determine whether the threshold for undue influence has been crossed. These include factors related to the influencer, characteristics and susceptibility of the testator, the nature of the relationship between the testator and the influencer, the circumstances under which the influence takes place, and the outcome of the proposed influence. Most of the existing literature on undue influence focuses on the “indicia” (meaning “signs” or “distinguishing features”) of undue influence, which are factors that appear to make undue influence more likely, though we are unaware of any studies which have demonstrated the validity and reliability of these factors.

Training as a medical expert in areas related to estate litigation and testamentary capacity is largely ignored in Canada. In the most recent Royal College of Physician and Surgeons Adult Neurology (2020), Adult Psychiatry (2020), and Geriatric Psychiatry (2018) training experiences, there is no mention of testamentary capacity, though the Geriatric Medicine Competencies (2019) do identify varying capacities, including the making of Wills and Testaments under assessment competencies. Acting as a medical expert in estate litigation has therefore been a role that has had to rely on the suboptimal “see one, do one, teach one” model of education, though there are increasing numbers of published primers^2^ and limited continuing education courses (involving both lawyers and medical experts) that are available for the interested learner. In this article, we will review the medical literature on undue influence, the conceptual models proposed for undue influence, and discuss the role of the medical expert in Will challenges which involve suspected undue influence. We will also briefly review some of the legal aspects related to undue influence. Most importantly, we will discuss the medical and psychiatric factors that could make individuals particularly susceptible to undue influence. Finally, we will propose what we consider to be the limited role of the medical expert in providing an opinion to the court related to undue influence.

## Methods

The authors were two lawyers specializing in estate litigation, and two academic geriatric psychiatrists with extensive experience acting as medical experts in estate litigation cases. The authors have previously worked together on legal cases and/or continuing legal and medical education programmes related to testamentary capacity/undue influence and estate litigation. The authors discussed a rationale and a specific outline for the article. The lawyers wrote a draft of the legal aspects section and the psychiatrists wrote the sections pertaining to the medical literature and the clinical aspects. The medical literature search utilized the Web of Science (WOS) with the search term “undue influence” and restriction to WOS categories of Law, Psychiatry, Ethics, and Psychology Clinical. All of the authors discussed and agreed on the role of the medical expert and the conclusions, and then revised and approved the final document.

### Undue Influence: Historical and Legal Issues

In common law, the onus is on the person challenging the Will to prove undue influence. The propounder (meaning the party that puts the Will forward for consideration) must demonstrate testamentary capacity, knowledge, approval, and due execution. However, as early as the 1800s, it was recognized that other factors might invalidate the Will. If a Will maker signed a Will “under coercion, or under the influence of fear, or in consequence of impressions created in his mind by fraudulent misrepresentations—in none of these cases can the instrument be properly described as being his will.”^6^ Furthermore, it has been noted that if the propounder is the sole or major beneficiary, the propounder must dispel the suspicious circumstances.^7^

But what kind of conduct amounts to undue influence? One of the first cases to address this was *Wingrove v. Wingrove* (1885).^8^ The conduct in question must “overcome the free will of the Will-maker.”^9^ The conduct could include force, threat of force, or other pressure. The frailty of a person impacts on the degree of force or pressure necessary to equate to undue influence.^10^ While the conduct may include actual violence, the threat of violence, and/or withholding care, it could also be a very ill person agreeing to anything just to get the influencer to stop. The conduct goes beyond simple persuasion and could include pleading, suggestions, urging or appeals. The conduct may also be fraudulent, which would include manipulation, telling lies, orchestrating isolation, use of threats.^11^ Importantly, the conduct destroys the free agency of the testator and constrains him to make a Will he would not have made in the absence of the influence.

More recently, the Wills, Estates, and Succession Act in 2014 in British Columbia addressed the issue of undue influence.^12^ It noted that if a person claims that a Will resulted from another person (a) being in a position where the potential for dependence or domination of the Will-maker was present and (b) was using that position to unduly influence the Will-maker to make the Will and establishes that the other person was in a position where the potential for dependence or domination of the Will-maker was present, the party seeking to defend the Will has the onus to prove that the person did not exercise undue influence over the Will-maker. This new provision provided that once a position of dominance or potential for dominance is established, the onus shifts to the recipient to prove the Will was not the product of undue influence.

In California, a state statute was enacted in 2014, which defined undue influence as excessive persuasion that causes another person to act or refrain from acting by overcoming that person’s free will and results in inequity.^13^ The statute requires judges to consider the vulnerability of the victim, the influencers’ authority, the actions or tactics used by the influencer, and the equity of the result.

Finally, the legal literature identifies “suspicious circumstances” as—circumstances which could involve the preparation of the Will, or circumstances tending to show that the free will of the testator was overborne by acts of coercion or fraud—which could shift the burden of proof in cases where a Will is contested. Because these suspicious circumstances are dealt with frequently in the medical literature as well, they will be discussed below.

### Conceptual Models and Screening Tools for Undue Influence

Several conceptual models have been used to describe undue influence (see Table 1). These models, developed by physicians, social workers, neuropsychologists and lawyers, have served as the basis for the development of screening tools and guidelines to be used in the identification and assessment of undue influence. For example, in response to the 2014 California statute mentioned above, Quinn et al. developed a screening tool for the California Adult Protective Services. The tool, developed from a literature review, focus groups, and interviews of experts, in the form of a checklist providing check boxes for a number of characteristics falling under 4 major areas: client vulnerability, influencer authority/position of power, actions or tactics, and unfair or improper outcomes.^14^ There are no studies that have assessed the validity and reliability of this checklist or any other formal assessment tool for undue influence.^5^

**Table 1.** Conceptual Models of Undue Influence.

Name	Elements
SCAM^15^	Susceptibility of victims, Confidential relationships between victim and abuser, Active procurement of assets, Monetary loss
IDEAL^16^	Isolation, Dependency, Emotional manipulation and/or Exploitation of a vulnerability, Acquiescence, Loss
CULT^17^	Keep person unaware, control time and environment, create dependency, suppress old and create new behaviours/attitudes, allow no criticism
UI Wheel^18^	Based on a domestic violence model
SODR^19^	Susceptibility of the victim, Opportunity for undue influence, Disposition to exert undue influence, Result
IPA Task Force^3^	Social and environmental risk factors, psychological and physical risk factors, legal risk factors

Undue influence has also been conceptualized as a form of elder abuse. In fact, one of the few studies to examine the prevalence of undue influence based on a community sample of over 2,000 older adult Canadians, determined that elder abuse occurred in about 40 per 1,000, and that “material abuse” was the most common form of abuse.^20^ Material abuse was defined as having anyone they knew who had taken any actions to obtain or use their funds, property, or other assets. Attempts to influence them to change their Will was one of 6 examples of material abuse and was reported by 0.4% of respondents. The abused were in poorer physical health which limited their activities, they were less likely to have someone to confide in, less likely to have someone to help them in the event of illness and had high levels of depression and suicidal ideation. In another study that attempted to address the prevalence of this type of abuse, over 10% of Irish Nursing Home staff reported seeing cases where they felt a resident who lacked capacity was visited by a solicitor or where a resident was placed under undue pressure to make a change to their Will.^21^ These authors did caution, however, that although some of the comments which were reported by staff were unequivocally coercive and abusive, the line between acceptable or reasonable attempts to persuade is not always clear.

### Risk Factors, Red Flags, Suspicious Circumstances, and Indicia of Undue Influence

Much of the published medical and legal literature deals with conditions and circumstances that have typically been associated with undue influence. These have been variously referred to as risk factors, red flags, suspicious circumstances, and indicia of undue influence. There are dozens of these factors which can be divided into those characteristics pertaining to the testator, the influencer, the relationship between the testator and the influencer, and the circumstances surrounding the making of the Will. Many of these factors have been summarized in Tables 2, 3, 4, and 5. While these factors have been recognized by the courts as being important in the determination of undue influence, they have neither been subjected to empirical studies regarding their prevalence and importance, nor do we know which of these factors are necessary or sufficient to suggest undue influence.

**Table 2.** Testator Characteristics.

Personal Characteristics of Testator	Circumstantial Characteristics of Testator
Greater age (>75 years old) Female > Male Unmarried/widowed/divorced Financially independent Mid-upper income levels Illiteracy Cultural subservience to an authority figure Taking multiple medications Frailty/multimorbidity Physical/medical factors (see text) Psychological/psychiatric factors (see text) Cognitive impairment (see text) Substance abuse	Living alone Recent loss of spouse/bereavement Social isolation Estranged from children Presence of family conflict Living in a remote location Cultural, religious, or language barriers

*Note*: See References 3, 22 to 24.

**Table 3.** Influencer Characteristics.

Male > Female History of current or past substance abuse Mental or physical health problems History of legal difficulties Socially isolated Unemployed and/or financial stressors Has a confidential or fiduciary relationship with testator Lives with testator Non-resident child More distant relative Formal or informal caregiver A “suitor”

*Note*: See references 3, 22–24.

**Table 4.** Characteristics of Relationship between Testator and the Alleged Influencer.

Testator is dependent on influencer (physically, psychologically) Influencer exploits testator’s vulnerabilities Influencer providing care and assistance with activities of daily living Influencer isolates testator from other friends and relatives Influencer threatens to withdraw care, attention, love Influencer threatens to institutionalize Influencer intimidates and deceives

*Note*: See references 3, 22–24.

**Table 5.** Circumstances Surrounding Making of the Will.

Radical change from previous will favouring influencer Frequent and/or unusual changes to will Influencer arranges for and attends appointments with lawyer Influencer speaks for or provides documents for testator Lawyer is unknown to testator Will executed on a death-bed Other legal changes e.g. POAs, deeds, *inter vivos* gifts

*Note*: See references 3, 22–24.

### The Role of the Medical Expert and Undue Influence

The routes to involvement in estate litigation vary. For the treating clinician, they may be asked to act primarily as a witness, testifying to their observations of their patient, and then possibly opining on testamentary capacity and susceptibility to undue influence, whether or not they were formally assessed. An external medical expert may also be requested to provide an opinion on testamentary capacity and susceptibility to undue influence either via a contemporaneous assessment (which would include a patient interview) or a retrospective (and often post-mortem) assessment based on a review of medical records.

The role of the medical expert in providing an opinion on undue influence, however, is controversial. From a legal perspective, the British Columbia Law Institute notes that when lawyers request an opinion from a medical expert about a testator’s capacity, they should appreciate that assessing susceptibility to undue influence is “not normally addressed” by medical experts.^23^ Should the lawyer specifically request this type of assessment, they should describe the legal concepts involved, and ask the medical expert to opine on the question “Does the Will-maker’s mental status impair his or her ability to make independent dispositive decisions despite pressure imposed by others?” In one of the earliest attempts to describe the role of the medical expert in assessing testamentary capacity, Spar and Garb^23^ make suggestions on how to provide testimony regarding undue influence. They suggest that testimony should cover: (1) the testator’s mental status and personality; (2) specific factors that could affect susceptibility to undue influence (including personality, function, cognitive deficits, physical and social circumstances); (3) the implications of these in the context of the indicia of undue influence, and discrepancies between the testator’s attitudes, goals and values; (4) provide a diagnostic impression. The International Psychogeriatric Association’s (IPA) Task Force on Testamentary Capacity and Undue Influence^3^ noted that the clinician’s role is to advise the court about a person’s vulnerability to undue influence, but it is the court that decides if undue influence actually occurred. While it is unclear from the recommendations whether the IPA believes the expert should document and testify as to the presence of the indicia of undue influence, the IPA clearly stresses the need to assess the risk factors/red flags of undue influence, going as far as to suggest that the greater the number of red flags, the more likely that undue influence occurred. Similarly, as part of the assessment process, the IPA recommends an examination of the Will-making patterns looking for changes in patterns of trust and expressed wishes with respect to the distribution of assets. More recently, Plotkin et al.^5^ suggest a somewhat different role for the medical expert regarding undue influence. While agreeing with the IPA about the determination of undue influence being the responsibility of the courts, they disagree with the assessment of Will-making patterns, which in their opinion is beyond the expertise of the medical expert. While they emphasize that the role of the medical expert should focus on the vulnerability of the testator (cognitive function, emotional and physical dependency), they also note that the medical expert can provide input into the influencer’s apparent authority (“…can the individual say “no” to the alleged influencer?”). Furthermore, while they suggest that less should be said about the influencer’s actions (including whether the influencer should have known about the testator’s vulnerability) medical experts can comment on the testator’s emotional reaction to the actions of the influencer.

We suggest that the medical expert must primarily focus on the susceptibility of the testator to undue influence based on specific cognitive, psychiatric, and physical function, as documented in the medical records and/or demonstrated in the clinical examination. This protects the expert from having to act as a detective or to opine on areas that are beyond their expertise, leaving those determinations of fact to the court. Experts must be careful not to be perceived as usurping the authority of the court. While in general, this de-emphasizes the need to review many of the indicia of undue influence, there may still be occasions when this is necessary. For example, in the case of retrospective assessments, there could be pertinent history provided in the medical records that might speak directly to the red flags. An example of this might be the social work and nursing notes from a hospitalization that document conversations between testator and the influencer about will-making and/or observed abuse and coercion.

As noted above, Plotkin et al.^5^ and the IPA^3^ disagree about the use of examining Will-making patterns for the purposes of providing an opinion on undue influence. It is unclear if noting whether the Will and beneficiaries have changed, and the number of changes over a specific period of time requires any specific expertise. It is clear, however, that such changes could be important in determining testamentary capacity and specifically whether the testator had the cognitive capacity to make and sustain a choice (often a function of intact executive function and/or memory). However, in the context of undue influence, we conceptualize a significant change in Will-making pattern to be one of the indica of undue influence, and perhaps one of the strongest indica. Will-making patterns can be considered indica of undue influence and still be left for ultimate determination by the courts, while the medical expert focuses more on susceptibility to undue influence.

### Clinical Determination of Susceptibility to Undue Influence

What are the symptoms and medical conditions that could potentially make someone more susceptible to undue influence and what is the relationship between vulnerability and the strength of undue influence required to interfere with the free will of the testator? Unfortunately, there are no studies that provide empirical answers to these questions. In the absence of such studies, however, medical experts can still provide valuable insights to the courts, derived from clinical experience.^24^

The medical expert will need to take an approach that considers both symptoms, as well as syndromes that lead to susceptibility. Broadly, these can involve: (a) cognitive function; (b) psychiatric symptoms; (c) physical function; and (d) behavioural function and addictions.

#### Cognitive function

Almost all types of cognitive deficits might increase susceptibility to undue influence either directly, by specifically impaired cognitive function, or indirectly, by increasing the testator’s dependence on the influencer. For example, impaired memory might make it easier for the influencer to convince the testator they had already agreed to Will changes, made promises about asset distribution, or deceive the testator about the lack of involvement and negative behaviours of other potential beneficiaries. Impaired language function or aphasia might impair understanding of communication related to the Will or might make the testator more isolated and reliant on the influencer. Impaired executive function might make the testator more susceptible because of the associated decrease in insight and judgement, and inability to assess competing claims including the sincerity, honesty and motivation of individuals in a position to exert influence.^5^ This could ultimately affect the ability of a testator to make and sustain a reasoned choice. Specific diagnoses of dementia, delirium (especially death bed Wills) and Mild Cognitive Impairment (MCI) help to document the presence of cognitive dysfunction, though there should still be an attempt to highlight the specific cognitive deficits and link them to susceptibility to undue influence. The medical expert can also help the court in the interpretation of scores on commonly used cognitive screening instruments.

#### Psychiatric symptoms

The presence of depression, bereavement, anxiety, and psychosis could all potentially increase susceptibility to undue influence. Depressive symptoms are often associated with loneliness, feelings of isolation, and negative views of oneself and the future. Moreover, these symptoms often occur in the early stages of dementia and MCI at a time when the cognitive changes are often not recognized. An influencer’s attention and promises of care can be very powerful in these situations. Suspiciousness and even overt paranoid delusions are also often signs of early dementia or MCI whereby individuals suffering from early cognitive changes either misperceive events and behaviours or attempt to compensate for memory deficits by paranoid explanations. These feelings are often directed against people close to the testator and with whom they may have had an ambivalent relationship thus influencing the disposition of their estate. In our experience, this is a common phenomenon in Will challenges. While persecutory ideation and paranoia might be protective against undue influence, it is also possible that the influencer could exploit these symptoms to turn the testator against other potential beneficiaries. The specific DSM diagnoses (e.g., Affective Disorders, Anxiety Disorders, Personality Disorders, Schizophrenia/Schizoaffective Disorder) will all help document the presence of psychiatric symptoms, but the medical expert should also attempt to explain how the specific symptoms associated with the disorder may increase susceptibility to undue influence. If a patient has significant suicidal ideation, they might not care about what happens to their estate in the context of an influencer pressuring them to make Will changes—they might do anything to get the influencer to leave them alone. Testators with Schizoid and Dependent Personality Disorders might be particularly susceptible to undue influence.^3^

#### Physical function

Impaired abilities to perform activities of daily living will make many testators more dependent on others for their care and in their ability to survive in their own homes. Physical characteristics such as vision and hearing as well as mobility play major roles in isolation and increasing dependency. Threats to institutionalize because of physical problems and frailty can be immensely powerful forms of influence for many vulnerable older individuals.

#### Behavioural function and addictions

Substance abuse can increase susceptibility to undue influence from both the neurotoxic effects of the drug as well as the dependence on an influencer to provide the addictive substance. Obtaining a regular supply of alcohol and/or cigarettes has long been a powerful potential influence for some testators, but more recently, access to a regular supply of medical marijuana is becoming an increasingly common scenario. Other potential behaviours that can be associated with undue influence include sexual bargaining.

In many cases, there are more than one of the factors described above, and the concept of multimorbidity should be considered when forming an opinion about susceptibility to undue influence. The notion of multimorbidity has also been characterized by Geriatric Medicine as “frailty syndrome” especially among the very old. Rockwood et al.^25^ have defined frailty as “a term widely used to denote a multi-dimensional syndrome of loss of reserves (energy, physical ability, cognition, health) that gives rise to vulnerability.” Finally, it is helpful for the medical expert to comment on the relationship between the number and severity of factors mentioned above and the relative force of the influence that would need to be exerted in order for it to be considered undue. For example, in the highly vulnerable individual with cognitive impairment, depression, and social isolation, the degree of influence necessary to be considered undue might be modest. In contrast, for the relatively healthy testator without physical, psychiatric, or cognitive dysfunction, the degree of influence necessary to be considered undue might be considerable.^26^ Even in the absence of red flags and factors which increase susceptibility to undue influence, a testator could still be influenced against their will, though this would require coercion or threats.

## Conclusions

The legal concept of undue influence can be used to challenge a Will. Given the changes in population demographics as well as added pressures from pandemics, it is anticipated that this will become an increasingly important aspect of estate litigation. While it is the responsibility of the court to determine if indue influence has occurred, we have argued that the medical expert can perform an important and useful function for the courts by focusing primarily on the susceptibility of the testator to undue influence. This susceptibility should be assessed based on evidence derived from the medical documentation, the medical examination, and the history. The medical expert should consider cognitive function, psychiatric symptoms in the mental status, as well as the physical and behavioural function of the testator in so far as such pertain to the susceptibility to undue influence. Medical experts must learn to accept a limited but important role in a medico-legal collaboration that facilitates the court’s ability to make the most informed decisions.
